# Two novel bombesin-like neuropeptides from the skin secretion of *Pelophylax* kl. *esculentus*: *Ex vivo* pharmacological characterization on rat smooth muscle types

**DOI:** 10.3389/fmolb.2022.953974

**Published:** 2022-09-29

**Authors:** Luyao Zhang, Chen Chen, Wanchen Zou, Xiaoling Chen, Mei Zhou, Chengbang Ma, Xinping Xi, Tianbao Chen, Chris Shaw, Mingchun Liu, Lei Wang

**Affiliations:** ^1^ Key Laboratory of Livestock Infectious Diseases in Northeast China, Ministry of Education, College of Animal Science and Veterinary Medicine, Shenyang Agricultural University, Shenyang, China; ^2^ School of Pharmacy, Queen’s University Belfast, Belfast, United Kingdom

**Keywords:** bombesin-like neuropeptides (BLPs), frog skin, molecule cloning, N-terminal variants, pharmacological properties, bombesin receptor, smooth muscle

## Abstract

Mammalian bombesin-like neuropeptides (BLPs) play an important role in regulation of physiological and pathophysiological processes. Frog skin-derived BLPs, of smaller size and diverse lengths and sequences at their N-terminus, have attracted the attention of many researchers. However, these N-terminal variants and the receptors modulating their pharmacological actions are poorly studied and less understood. In this study, two BLPs, namely, [Asn^3^, Lys^6^, Thr^10^, Phe^13^]3–14-bombesin and [Asn^3^, Lys^6^, Phe^13^]3–14-bombesin with primary structures NLGKQWATGHFM and NLGKQWAVGHFM were isolated from the skin secretion of hybrid *Pelophylax* kl. *esculentus*. Both BLPs share a similar primary structure with only a single amino acid substitution at the eighth position (threonine to valine), while they have quite different myotropic potencies with EC_50_ values in the range of 22.64 ± 9.7 nM (*N* = 8) to 83.93 ± 46.9 nM (*N* = 8). The potency of [Asn^3^, Lys^6^, Thr^10^, Phe^13^]3–14-bombesin was approximately 3-fold higher than that of [Asn^3^, Lys^6^, Phe^13^]3–14-bombesin. Through the investigation of receptor selectivity using a canonical bombesin receptor antagonist, it was found that [Asn^3^, Lys^6^, Thr^10^, Phe^13^]3–14-bombesin and [Asn^3^, Lys^6^, Phe^13^]3–14-bombesin had an affinity to both BB1 and BB2 receptors. Their contractile functions are mainly modulated by both BB1 and BB2 receptors on rat urinary bladder and BB2 alone on rat uterus smooth muscle preparations. These data may provide new insights into the design of potent and selective ligands for bombesin receptors. Moreover, [Asn^3^, Lys^6^, Thr^10^, Phe^13^]3–14-bombesin and [Asn^3^, Lys^6^, Phe^13^]3–14-bombesin did not induce significant hemolysis and toxicity in normal human cells, suggesting that these two natural novel BLPs have great potential for development into new drug candidates.

## 1 Introduction

Neuropeptides drive a broad range of biological actions and play a major role in regulating multiple functions involving all organ systems in mammals. Amphibian skin-derived neuropeptides, including bombesin, bradykinin, litorin, caerulein, tachykinin, tryptophyllin, dermorphin, and deltorphin, have been considered part of their host-defense system and display a wide range of biological effects on various physiological processes (Pukala et al., 2006a; Bilusich et al., 2009). Bombesin-like peptides (BLPs), derived from amphibians, which function *via* activating G-protein-coupled receptors, have been shown to modulate multiple pharmacological effects on smooth muscles in various organs, including blood vessels, the stomach, intestines, the bladder, airways, and the uterus (Bardin, 1993; Williams, 2015).

Gastrin-releasing peptides (GRP) and neuromedin-B (NMB) are two BLPs found in mammals (Jensen et al., 2008). Originally isolated from porcine stomach, GRP stimulates gastrin release, smooth muscle contraction, and proliferation of epithelial cells (Hirooka et al., 2021). NMB was latterly identified from porcine tissue and has been found to contract smooth muscles, influence endocrine secretion, and regulate blood pressure (Hiroko, 2000). The amphibian counterparts of GRP and NMB have been found in frog skins, which are bombesin and ranatensin/litorin (Gonzalez et al., 2008). Unlike mammalian BLPs, frog skin-derived BLPs show considerable diversity in their sequences at the N-terminus, while normally retaining a highly conserved sequence at the C-terminus. Thus, they have been classified into three families according to the sequence in their conserved domains. Their C-terminal-conserved sequence is GHXM, where X is either L in the bombesin/alytesin family or F in the ranatensin family or both are in the phyllolitorin family (Spindel et al., 1993; Spindel and Kastin, 2006). Additionally, C-terminal amination is a common feature of amphibian bombesin-related peptides, which is crucial for high-affinity binding of the peptide with its receptor (Gonzalez et al., 2008).

To date, there are three types of bombesin receptors, which are BB1, BB2, and an orphan receptor, BB3, found in mammals (Ramos-Alvarez et al., 2015). The previous studies on bombesin receptors suggested that the BB1 receptor, also known as the NMB receptor, was mainly distributed in the mammalian esophagus, intestines, testis, and urinary bladder (Spindel et al., 1993; Ohki-Hamazaki et al., 2016). In addition to the stimulatory functions on smooth muscle, the activation of the BB1 receptor also influences the CNS and tumor growth (Gonzalez et al., 2008). The tumor-related BB2 receptor, a GRP-preferring receptor, was primarily expressed in the pancreas, while the expression levels in the colon, breast, prostate, and some regions of the CNS were relatively low (Baratto et al., 2020). The BB3 receptor has been found to regulate food intake, metabolic rate, body weight, and insulin release; however, it had low affinities to both GRP and NMB and all naturally occurring BLPs. No endogenous or natural ligand for BB3 has been identified so far (Gonzalez et al., 2008; Ladenheim and Kastin, 2013). The lack of a ligand has impeded the study of BB3 as it is challenging to establish BB3 involvement in physiological and pathophysiological processes. Therefore, the pharmacology of BB3 remains unclear and largely unstudied.

Recently, the discovery of novel natural neuropeptides and exploring the mechanisms of their function have emerged as a hot research topic as they have great potential as future therapeutics. Here in this study, two novel BLPs, [Asn^3^, Lys^6^, Thr^10^, Phe^13^]3–14-bombesin and [Asn^3^, Lys^6^, Phe^13^]3–14-bombesin, have been isolated and identified from the skin secretion of the hybrid frog, *Pelophylax* kl. *esculentus*. Isolated smooth muscle tissues, due to their autonomic-related functions, provide a sketch of a pharmacophore model for studying these ligands and their target cell–surface receptors. Therefore, the pharmacological activities of these two BLPs and possible receptors which mediate their functions were investigated using rat *ex vivo* smooth muscle preparations.

## 2 Materials and methods

### 2.1 Acquisition of *Pelophylax* kl. *esculentus* skin secretion

Adult *Pelophylax kl. esculentus* frogs were obtained commercially and conditioned for 4 months before secretion harvesting. They were housed at 20°C–25°C under a 12-h light/dark cycle. The dorsal skin surface was stimulated by gentle transdermal electrical stimulation (3–5 V, 100 Hz). The skin secretion was washed from the skin using deionized water, snap-frozen in liquid nitrogen, lyophilized, and stored at −20°C prior to analysis. The lyophilized secretion in this study was utilized as the sample for both mRNA isolation (molecular cloning sequencing) and crude material fractionation sequencing with Liquid Chromatography Quadrupole ion trap Mass Spectrometry (LCQ MS). All protocols involving animal subjects in this study were conducted in accordance with the UK Animal (Scientific Procedures) Act 1986, approved by the Institutional Animal Care and Use Committee of Queen’s University Belfast on 1st March 2011. All procedures were conducted under the authority of the Project License PPL 2694, issued by the Department of Health, Social Services, and Public Safety, Northern Ireland.

### 2.2 Molecular cloning and nucleotide sequencing

The degenerate sense primer (S1; 5′-GAWYYAYYHRAGCCYAAADATG-3′) in 3′-Rapid amplification of cDNA end polymerase chain reaction (RACE PCR) was designed to a highly conserved domain of the 5′-untranslated region of previously characterized skin peptide-encoding cDNAs from closely related *Rana* species. The primer annealing temperature of the PCR cycling procedure was 54°C. Poly A mRNA isolation from *Pelophylax* kl. *esculentus* skin secretion was achieved by application of a Dynabeads^®^ mRNA DIRECT™ Kit (Dynal Biotech Ltd., Bromborough, UK). Both 5′ and 3′rapid amplifications of the cDNA ends (RACE) were performed by using the BD SMART™ RACE cDNA Amplification Kit (BD Biosciences Clontech, Palo Alto, CA, United States). PCR product purifications were performed using an E.Z.N.A. ^®^ Cycle Pure Kit (Omega Bio-Tek Inc., Norcross, GA, United States). pGEM®-T Easy Vector (Promega, Madison, WI, United States), 2 × rapid ligation buffer (Promega), and T4 DNA Ligase (3 Unit/µl, Promega) were used in the ligation procedure. JM109 high-efficiency *E. coil* competent cells (>10^8^ CFU/μg, Promega, Madison WI, United States) were used for transformations. The cloning PCR reaction was performed using an Advantage^®^ 2 polymerase Mix (Clontech Laboratories, Inc., Mountain View, CA, United States) and Advantage^®^ 2 PCR Kit (Clontech). After the sequencing reaction using the Big Dye^®^ Terminator v 3.1 Cycle Sequencing Kit (Applied Biosystems, Rotkreuz, Switzerland) and ethanol purification, the extension product was sequenced by use of an ABI 3730 automated sequencer (Applied Biosystems, Foster City, CA, United States).

### 2.3 Fractionation of frog skin secretion and peptide sequencing

The lyophilized frog secretion was dissolved in deionized water and then vortexed for 20 min and centrifuged at 20,000 × g for 5 min. The supernatant was filtered and clarified and then slowly injected into the reverse-phase high-performance liquid chromatography (RP-HPLC) system and fractionated using gradient elution. The gradient of the elution program of RP-HPLC was set from 0.05/99.95 (v/v) TFA/water (0 min) to 0.05/19.95/80.0 (v/v/v) TFA/water/acetonitrile over 240 min (UV Detector: Waters 2489; Binary HPLC Pump: Waters 1525; Dublin, Ireland). The column (Jupiter C5 300 Å column 250 mm × 4.6 mm) was eluted at a flow rate of 1 ml/min, and the effluent was continually monitored at a UV wavelength of 214 nm. Meanwhile, the eluted components of every peak were collected into tubes. The RP-HPLC fractions were injected into a liquid chromatography mass spectrometry (LC-MS) system (Thermo Fisher Scientific, San Francisco, CA, United States) in the positive detection mode. The capillary of electrospray ionization (ESI) was heated at 320°C, and the voltage of the spray electric field was set at 4.5 kV. After that, the sample was ionized by an ESI probe and transferred through the quadrupole mass filter until reaching the ion-trap mass analyzer. Helium was utilized as the buffer gas in the ion trap, and it also served as an activation partner in the collision-induced dissociation for tandem mass spectrometry (MS/MS) sequencing. The Thermo Scientific Proteome Discoverer 1.0 software Sequest algorithm was used to analyze the resultant data.

### 2.4 Synthesis and purification of [Asn^3^, Lys^6^, Thr^10^, Phe^13^]3–14-bombesin and [Asn^3^, Lys^6^, Phe^13^]3–14-bombesin

[Asn^3^, Lys^6^, Thr^10^, Phe^13^]3–14-bombesin and [Asn^3^, Lys^6^, Phe^13^]3–14-bombesin were synthesized by using the solid-phase peptide synthesis technique through an automated solid phase 2-channel peptide synthesizer (Tribute^®^, Gyros Protein Technologies, United States) along with Rink Amide MBHA resin (100–200 mesh) and Fmoc chemistry. An optimized cleavage cocktail was developed for removing the peptides from resin and side chain-protecting groups as described elsewhere (Marquez et al., 2000). The synthetic peptides were lyophilized instantly after being washed using cold diethyl ether. Finally, each synthetic peptide was purified using RP-HPLC (Phenomenex C-5 column, 0.46 cm × 25 cm). The collected fractions were reserved and analyzed by LCQ™-Fleet electrospray ion-trap mass spectrometry (Thermo Fisher Scientific, San Francisco, CA, United States) and RP-HPLC to verify the molecular masses and the purity (>90 %) of the target peptides (Supplementary Figures S1, S2). The HPLC fractions containing the purified peptide of interest were lyophilized and subsequently subjected to nuclear magnetic resonance spectroscopy (NMR). The ^1^H-NMR spectrum (Supplementary Figure S3) of each pure peptide (6 mM in deuterium oxide) was recorded on a Bruker Ultrashield 400 MHz spectrometer (Bruker, Coventry, UK) at 25°C.

### 2.5 Assessment of cellular toxicity

Hemolysis and lactate dehydrogenase (LDH) assays were employed for initial assessment of the cellular toxicity of [Asn^3^, Lys^6^, Thr^10^, Phe^13^]3–14-bombesin and [Asn^3^, Lys^6^, Phe^13^]3–14-bombesin. Horse erythrocyte (TCS Biosciences Ltd., Buckingham, UK) suspension (4 %) was used in the hemolysis activity test. Peptides were dissolved in PBS buffer and diluted into a range of concentrations from 256 to 2 μM. The 4 % suspension of the erythrocytes was incubated with an equivalent volume of the peptide at 37°C for 2 h. Equivalent horse red blood cells treated with phosphate-buffered saline (PBS) or 1 % Triton X-100 served as the negative and positive controls, respectively. After incubation, the mixture of peptide and horse erythrocytes was centrifuged at 930 × g for 5 min before measurement of absorbance at 550 nm in supernatants using a Synergy HT plate reader (BioTek Instruments, Inc., Winooski, VT, United States).

The cytotoxic effect of peptides on human microvascular endothelial cells (HMEC-1, purchased from the American Type Culture Collection) was measured using the CyQuant™ LDH Cytotoxicity Assay Kit (Thermo-Fisher Scientific, Oregon, United States) according to the manufacturer’s instruction. HMEC-1 cells were grown in the molecular, cellular, and development biology 131 (MCDB-131) medium (supplemented with 15 % fetal bovine serum, 10 ng/ml epidermal growth factor, 10 mM glutamine, and 1 μg/ml hydrocortisone) until a cell layer of 80 % confluence was established. Cells were seeded into a 96-well plate at a density of 10,000 cells/well. After incubation for 24 h at 37°C with 5 % CO_2_, the cells were starved for 4–6 h by replacing the medium with serum-free medium. Thereafter, for the synthesized peptides, concentrations ranging from 10^−9^ M to 10^−4^ M in the serum-free medium were incubated with the cells for 24 h. Equivalent cells treated with sterile, deionized water (1 %) or lysis buffer (1 %) (supplied in the kit) served as the negative and positive controls, respectively. Then, 50 μl of the supernatant from each well was mixed with an equal volume of the supplied reaction mixture and incubated at room temperature. After 30-min incubation, the reactions were stopped by adding 50 μl of the stop solution supplied in the kit. The level of LDH released from damaged cells was measured using a Synergy HT plate reader at absorbance values of 490 and 680 nm.

### 2.6 Pharmacological assay of [Asn^3^, Lys^6^, Thr^10^, Phe^13^]3–14-bombesin and [Asn^3^, Lys^6^, Phe^13^]3–14-bombesin on rat bladder and uterus smooth muscles

#### 2.6.1 Tissue preparation

Urinary bladder and uterus were isolated from adult female Wistar rats (250–300 g). The rats were killed by asphyxiation with CO_2_ followed by cervical dislocation under UK Animal Research licenses. The animals were laid on their dorsal surface, and the fur was trimmed to expose the abdominal muscle. Subsequently, the abdominal muscle was cut at the mid-ventral line from the bottom, and the whole bladder and uterus were removed carefully. Upon removal of the intact bladder and whole uterus from the animals, they were placed in ice-cold Kreb’s solution (118 mM NaCl, 1.15 mM NaH_2_PO_4_, 2.5 mM CaCl_2_, 25 mM NaHCO_3_, 4.7 mM KCl, 1.1 mM MgCl_2_, and 5.6 mM glucose) immediately. They were then cut to an appropriate size to be mounted on the transducers (AD Instruments Pty Ltd., UK).

#### 2.6.2 Pharmacological assays

Pharmacological experiments in this study were performed on an isometric contractile apparatus. The peptides used were dissolved and prepared into stock solutions using Kreb’s solution. The fresh working solutions were prepared on the day of the experiments by diluting stock solutions using Kreb’s solution. The dose responses were then performed and recorded following applications of peptides in the range of 10^−11^–10^−5^ M to each organ bath. The changes in the muscle tone and frequency of contractions produced by the peptides could be identified through comparison with spontaneous contractions prior to peptide application.

The examination of bombesin receptor selectivity of peptides on rat urinary bladder and uterus smooth muscle was performed by using a BB1 receptor antagonist (3 × 10^−7^ M, BIM 23042, Tocris Bioscience, Bristol, UK) and BB2 receptor antagonist (10^−6^ M, BW2258U89, Phoenix Pharmaceuticals, California, United States), which were applied to tissue preparations prior to peptide treatment.

### 2.7 Statistical analysis

Data were analyzed by Graphpad Prism software 6.0 (San Diego, United States) to establish comparative EC_50_ values (the concentration required to induce half-maximal response) for each peptide, and dose–response curves were constructed using a best-fit algorithm through the data analysis package provided. Responses were plotted as tension changes of contraction or changes in the contractile frequency against the final molar concentration of peptides in the organ bath. Data points represented mean values ± SEM (standard error mean), and sample size (n) referred to the number of replicates.

## 3 Results

### 3.1 [Asn^3^, Lys^6^, Thr^10^, Phe^13^]3–14-bombesin and [Asn^3^, Lys^6^, Phe^13^]3–14-bombesin precursor-encoding cDNAs

The full length of the coding sequences and amino acid sequences of the [Asn^3^, Lys^6^, Thr^10^, Phe^13^]3–14-bombesin and [Asn^3^, Lys^6^, Phe^13^]3–14-bombesin precursors (Supplementary Figure S4) were identified from the skin secretion of *Pelophylax* kl. *esculentus* through molecular cloning and Sanger dideoxy sequencing. The precursor of [Asn^3^, Lys^6^, Thr^10^, Phe^13^]3–14-bombesin had a very close similarity to the [Asn^3^, Lys^6^, Phe^13^]3–14-bombesin precursor but were subject to five amino acid substitutions, while their its encoding nucleotide sequence exhibited ten base differences (Supplementary Figure S5). Both BLP precursors possessed an open-reading frame of 88 amino acids, comprising an N-terminal signal peptide with 29 amino acids in length, a pro-peptide cleavage site (-Leu-Arg-), and a single copy of a mature peptide with C-terminus terminating in a glycyl residue amide donor and a further pro-peptide cleavage site (-Lys-Arg-). The sequences of their mature peptide regions shared very similar structures with a single amino acid substitution at the eighth position (threonine to valine). Based on their highly conserved mature peptide sequences and the C-terminal motif ending in -GHFM-amide, these two peptides were classified into a BLP family, ranatensin. The seven amino acid residues at the C-terminus of the peptide [Asn^3^, Lys^6^, Thr^10^, Phe^13^]3–14-bombesin was identical to those of pig NMB and human NMB-32, while the seven-amino acid region of the peptide [Asn^3^, Lys^6^, Phe^13^]3–14-bombesin was identical to that of ranantensin and human GRP. The cDNA sequences of these two BLPs have been deposited in the NCBI database, the accession code for [Asn^3^, Lys^6^, Thr^10^, Phe^13^]3–14-bombesin and [Asn^3^, Lys^6^, Phe^13^]3–14-bombesin are AXC59784 and MZ450144, respectively.

### 3.2 Primary structural confirmation of [Asn^3^, Lys^6^, Thr^10^, Phe^13^]3–14-bombesin and [Asn^3^, Lys^6^, Phe^13^]3–14-bombesin from frog skin secretion

**FIGURE 1:**
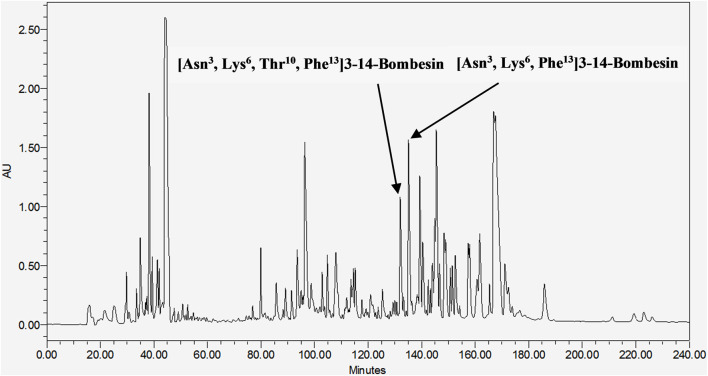
RP-HPLC chromatogram of *Pelophylax* kl. *esculentus* skin secretion (240 min). The retention time of [Asn^3^, Lys^6^, Thr^10^, Phe^13^]3–14-bombesin and [Asn^3^, Lys^6^, Phe^13^]3–14-bombesin is indicated by arrows.

These two mature peptides, [Asn^3^, Lys^6^, Thr^10^, Phe^13^]3–14-bombesin and [Asn^3^, Lys^6^, Phe^13^]3–14-bombesin, were subsequently isolated from the frog skin secretion of *Pelophylax* kl. *esculentus* through chromatographic fractionation (Figure 1), and their primary sequences were elucidated by MS/MS fragmentation sequencing (Supplementary Figure S6). [Asn^3^, Lys^6^, Thr^10^, Phe^13^]3–14-bombesin was contained in the fraction eluted at 133 min, while [Asn^3^, Lys^6^, Phe^13^]3–14-bombesin was eluted later at 135 min. These results confirmed that both BLP-encoding genes were activated and co-expressed in the frog skin glands.

### 3.3 Cellular toxicity of peptides on horse erythrocytes and the HMEC-1 cell line

**FIGURE 2:**
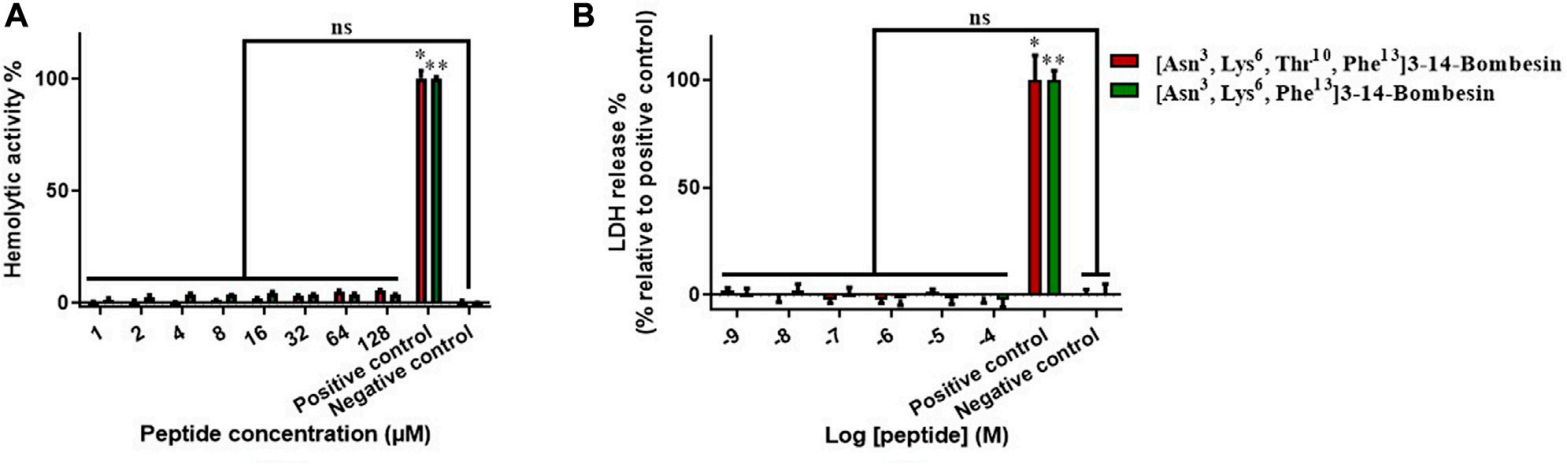
Detection of the cellular toxicities of [Asn^3^, Lys^6^, Thr^10^, Phe^13^]3–14-bombesin and [Asn^3^, Lys^6^, Phe^13^]3–14-bombesin. **(A)** Hemolysis of peptides on horse erythrocytes after 2 h of incubation. **(B)** Percentage of LDH release from HMEC-1 cells treated with peptides for 24 h; Error bar represents standard error mean (SEM) from three individual experiments, nine replicates. Statistical analysis was performed using the Kruskal–Wallis test in one-way ANOVA, (*p < 0.05, **p < 0.01 versus negative control). NS stands for not significant.

The cellular toxicity of [Asn^3^, Lys^6^, Thr^10^, Phe^13^]3–14-bombesin and [Asn^3^, Lys^6^, Phe^13^]3–14-bombesin was preliminarily investigated on horse red blood cells and a human normal cell line, HMEC-1 (Figure 2). There was no significant hemolysis induced by these two BLPs at the tested concentrations (Figure 2A). In the LDH cytotoxicity assay, the result (Figure 2B) from the 24-h exposure to various concentrations from 1 nM to 100 µM indicated that [Asn^3^, Lys^6^, Thr^10^, Phe^13^]3–14-bombesin and [Asn^3^, Lys^6^, Phe^13^]3–14-bombesin did not damage normal human cells.

### 3.4 Pharmacological tests of peptides on rat bladder and uterus

#### 3.4.1 Bladder

**FIGURE 3:**
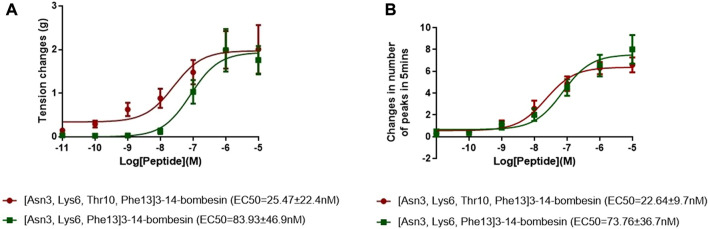
Dose–response curves of [Asn^3^, Lys^6^, Thr^10^, Phe^13^]3–14-bombesin (red) and [Asn^3^, Lys^6^, Phe^13^]3–14-bombesin (green) using **(A)** rat urinary bladder and **(B)** rat uterus smooth muscle preparations. Each point represents the mean of eight replicates, which are measurements of individual tissue preparations on different days, and bars represent the SEM.

**FIGURE 4:**
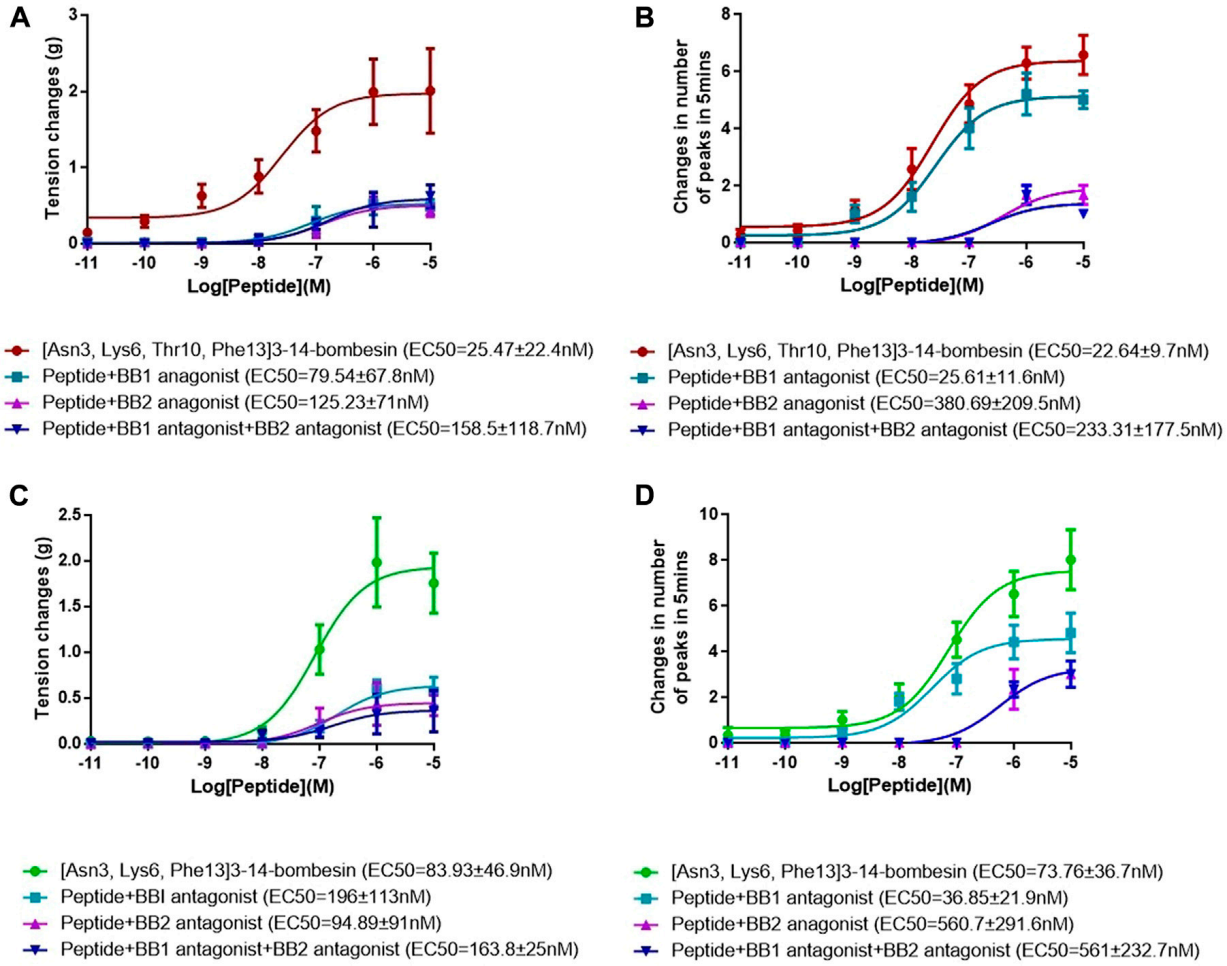
Dose–response curves of [Asn^3^, Lys^6^, Thr^10^, Phe^13^]3–14-bombesin in the presence and absence of the BB1 antagonist and BB2 antagonist using **(A)** rat urinary bladder and **(B)** rat uterus smooth muscle preparations. Dose–response curves of [Asn^3^, Lys^6^, Phe^13^]3–14-bombesin in the presence and absence of BB1 antagonist and BB2 antagonist using **(C)** rat urinary bladder and **(D)** rat uterus smooth muscle preparations. Each point represents the mean of six replicates, which are measurements of individual tissue preparations on different days, and error bars represent SEM.

**FIGURE 5:**
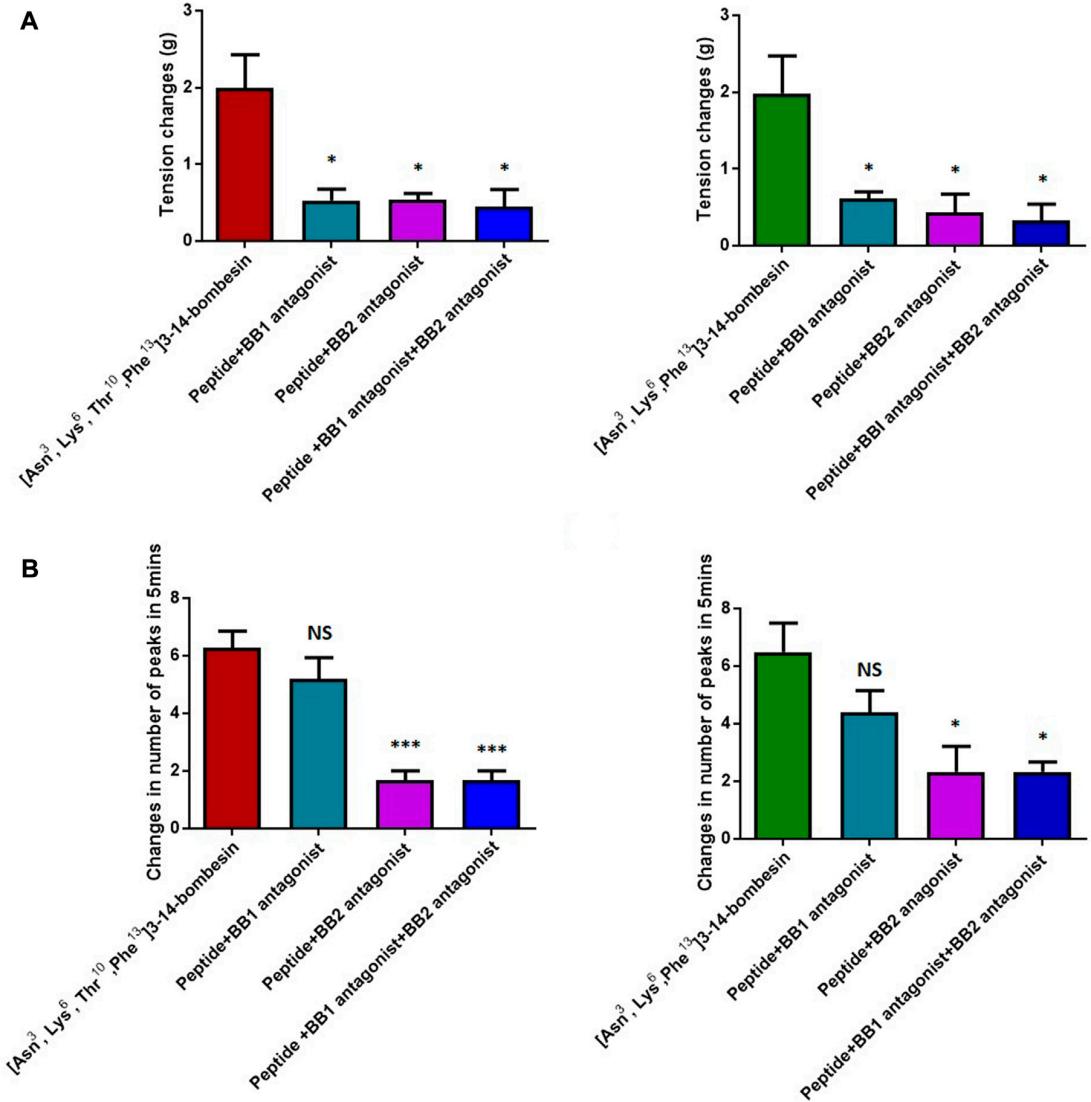
Contractile responses of [Asn^3^, Lys^6^, Thr^10^, Phe^13^]3–14-bombesin (1 μM) and [Asn^3^, Lys^6^, Phe^13^]3–14-bombesin (1 μM) on various smooth muscle preparations in the presence and absence of BB1 and BB2 receptor antagonists. **(A)** Rat urinary bladder smooth muscle preparations; **(B)** rat uterus smooth muscle preparations. Statistical significance of the difference was analyzed by using one-way ANOVA (*p < 0.05, ***p < 0.001), NS means not significant. Each error bar represents the SEM of six repeats which are measurements of individual tissue preparations on different days.

The dose–response curves of [Asn^3^, Lys^6^, Thr^10^, Phe^13^]3–14-bombesin and [Asn^3^, Lys^6^, Phe^13^]3–14-bombesin on rat bladder are shown in Figure 3A. Both peptides significantly enhanced the spontaneous contraction of the rat urinary bladder. The EC_50_ values were calculated as 25.47 ± 22.4 nM (*N* = 8) and 83.93 ± 46.9 nM (*N* = 8), respectively. For the examination of bombesin receptor selectivity, BB1 and BB2 receptor antagonists were applied alone and in combination on the rat bladder smooth muscle as pre-treatment. The dose–response curves of peptides with and without the receptor antagonists are shown in Figures 4A , C, 5A. Specifically, both BB1/NMB receptor antagonist (BIM 23042) and BB2/GRP receptor antagonist (BW2258U89) significantly inhibited the [Asn^3^, Lys^6^, Thr^10^, Phe^13^]3–14-bombesin- and [Asn^3^, Lys^6^, Phe^13^]3–14-bombesin-inducing contractile responses on rat bladder (Figures 4A, C, 5A), indicating that the contractile functions of both BLPs on the rat bladder were regulated by both BB1 and BB2 receptors (Marquez et al., 2000; Blais et al., 2016). The EC_50s,_ the measure of the potency of both BLPs in the presence of the receptor antagonists, were greater than those in the absence of the receptor antagonists, revealing that both BB1 and BB2 receptor antagonists significantly reduced the potencies of these two BLPs on the rat bladder. This maybe because canonical antagonists occupied a certain number of BB1 and BB2 receptors and stopped these receptors from binding to BLPs and producing a response. The potencies of these two BLPs are mainly determined by the number of empty receptors available and their affinity to the receptors. When the relationship between receptor occupancy and response is linear, the dissociation constant KD, a parameter characterizing the affinity of an agonist to the receptor, equals EC_50_ (Salahudeen and Nishtala, 2017). To investigate the detailed characterization of these two BLPs against BB1/2/3 receptors, some *in vitro* studies are underway in the laboratory.

In addition, it was observed that the combined pre-treatment of BIM 23042 and BW2258U89 showed no significant difference from a single application of either of them (Figure 5), suggesting that both BLPs might exert their functions through BB1 and BB2 receptors. Also, in the presence of both BIM 23042 and BW2258U89, these two BLPs at a concentration of 1 μM could partially maintain the contractile activity on the rat bladder (Figure 5A). It suggested that some other types of receptors (orphan bombesin receptors) were likely involved in the contractile response to [Asn^3^, Lys^6^, Thr^10^, Phe^13^]3–14-bombesin and [Asn^3^, Lys^6^, Phe^13^]3–14-bombesin.

#### 3.4.2 Uterus

Both [Asn^3^, Lys^6^, Thr^10^, Phe^13^]3–14-bombesin and [Asn^3^, Lys^6^, Phe^13^]3–14-bombesin significantly increased the spontaneous contraction of rat uterus in a dose-dependent manner. The corresponding dose–response curves are shown in Figure 3B. The EC_50_ values of both BLPs were calculated as 22.64 ± 9.7 nM (*N* = 8) and 73.76 ± 36.7 nM (*N* = 8), respectively. BB1 and BB2 receptor antagonists were applied alone and in combination on rat uterus smooth muscle as pre-treatment to examine bombesin receptor selectivity of the two BLPs. The dose–response curves of peptides with and without receptor antagonists are shown in Figures 4B , D, 5B.

Unlike the result from the test with rat bladder, the BB1/NMB receptor antagonist did not demonstrate considerable attenuating contractile effects on either [Asn^3^, Lys^6^, Thr^10^, Phe^13^]3–14-bombesin or [Asn^3^, Lys^6^, Phe^13^]3–14-bombesin in the rat uterus. In contrast, the BB2/GRP receptor antagonist significantly reduced the effect, indicating that both BLPs exerted their contractile function on rat uterus smooth muscle preparations mainly through the BB2/GRP receptor. This phenomenon might be caused by the difference in receptor distribution between the rat urinary bladder and uterus, and the intensity of the BB1/NMB receptor expressed on the rat urinary bladder is higher than that on the rat uterus. Kilgore et al. previously revealed that the rat bladder mainly expresses the NMB receptor, and the rat uterus expresses the GRP receptor through radioligand-binding studies (Kilgore et al., 1993).

## 4 Discussion

Frog skin secretions have provided a rich source of biologically active neuropeptides that are similar to mammalian peptides (Pukala et al., 2006b). Compared to the parental species, the hybrid amphibian system was believed to have greater peptide diversity or even strengthened biological activity. For instance, apart from the libraries of peptides that occur in both parental taxa, four peptides that are not expressed by the parent species have been reported from the hybrid tree frogs of *Litoria splendida* and *Litoria caerulea* (Daum et al., 2012). Daum et al. revealed that the skin secretion from the hybrid frog species, *Pelophyla*x kl. *esculentus*, could manifest a more potent fungicidal effect against *Batrachochytrium dendrobatidis*, along with a greater antimicrobial peptide diversity (Hirooka et al., 2021). However, the hybrid amphibian systems are largely unstudied. Therefore, this study was designed to focus on “shotgun cloning” of novel BLPs from the hybrid tree frog by using a degenerate primer and evaluating the bioactivity. BLPs from amphibians are homologous counterparts of endogenous mammalian neuropeptides that regulate central homeostatic mechanisms in mammals (Spindel, 2013). They have been divided into three families according to their structures, which are bombesin, ranatensin, and litorin families. In mammals, gastrin-releasing peptide (GRP) belongs to the bombesin family, and NMB belongs to the ranatensin family (Battey et al., 2021). They have a stimulant effect on smooth muscle contraction and influence a wide range of biological processes, including thermoregulation, itch sensation, cell growth, secretions, blood pressure regulation, heart rate control, food intake, and digestion (Battey et al., 2021). Therefore, it is essential to understand their physiological and pathological relevance as these could provide new insights into their associated diseases.

Herein, through molecular cloning and DNA sequencing techniques, along with RP-HPLC and MS/MS fragmentation technology of LCQ mass spectrometry, two novel BLPs, namely, [Asn^3^, Lys^6^, Thr^10^, Phe^13^]3–14-bombesin and [Asn^3^, Lys^6^, Phe^13^]3–14-bombesin with contractile functions on the rat smooth muscle of the urinary bladder and uterus were identified from the skin secretion of *Pelophylax* kl. *esculentus*. Structurally, [Asn^3^, Lys^6^, Thr^10^, Phe^13^]3–14-bombesin differed from [Asn^3^, Lys^6^, Phe^13^]3–14-bombesin with a single amino acid at the eighth position. Based on the sequence of the canonical tetrapeptide (-GHFM-amide) at the C-terminal regions of [Asn^3^, Lys^6^, Thr^10^, Phe^13^]3–14-bombesin and [Asn^3^, Lys^6^, Phe^13^]3–14-bombesin, they were initially classified into the ranatensin family of BLPs. This family shares a phenylalanine residue at the second position from the C-terminus, which is distinct from leucine in the bombesin family. Smooth muscle contraction effects on the bladder, uterus, and ileum preparations have been previously reported for the members of the ranatensin family (Anastasi et al., 1975).

There are three native bombesin-related peptides and their encoding mRNAs that have been found so far in the frog, *Pelophyla*x kl. *esculentus*, including bombesin-PE, which has been described in a previous study (Marenah et al., 2004). In this study, the nucleotide sequences and amino acid sequences of the open-reading frame encoding the precursors of both [Asn^3^, Lys^6^, Phe^13^]3–14-bombesin and [Asn^3^, Lys^6^, Thr^10^, Phe^13^]3–14-bombesin were reported. They have a high similarity to each other, and they contain only five amino acid substitutions in their amino acid sequences of entire open-reading frames, while there are ten base differences in their encoding nucleotide sequences. It is not surprising that a single frog species could express different mRNAs to encode variable BLPs, even with a highly conserved sequence. The frog skin secretion of *Bombina variegata*, for example, was proven to have at least four bombesin-related peptides and an exact copy of bombesin (Bai et al., 2012; Ramos-Alvarez et al., 2015), in which, bombesin-like-peptide-1 and canonical bombesin have only one amino acid different at the sixth position residue from histidine to asparagine (Bai et al., 2012). This phenomenon implies that a single species may contain diverse molecules that most likely result from the associated effects of gene mutations and duplications. As the universal occurrence of neuropeptides in the frog skin was considered to participate in skin defensive responses against predators (Davis et al., 1992) and different forms of BLPs might have varying half-life (Fleischmann et al., 2005), the frogs need more complicated cocktails in skin secretion that makes predators or pathogens more difficult to fight against them and protects them from attack or invasive microorganisms to some extent.

In terms of myotropic function, both BLPs [Asn^3^, Lys^6^, Thr^10^, Phe^13^]3–14-bombesin and [Asn^3^, Lys^6^, Phe^13^]3–14-bombesin showed similar pharmacological activities with different potencies, whereas peptides also had several distinct functional features. They exhibited great contractile effects on either the rat urinary bladder or uterus smooth muscles with different potencies, which were determined by EC_50_ values of 22.64 ± 9.7 nM (*N* = 8) and 73.76 ± 36.7 nM (*N* = 8) on the rat uterus, along with 25.47 ± 22.4 nM (*N* = 8) and 83.93 ± 46.9 nM (*N* = 8), respectively, on the urinary bladder. [Asn^3^, Lys^6^, Thr^10^, Phe^13^]3–14-bombesin was more potent than [Asn^3^, Lys^6^, Phe^13^]3–14-bombesin in evoking both smooth muscle tissues. The potency of [Asn^3^, Lys^6^, Thr^10^, Phe^13^]3–14-bombesin seemed to be approximately increased 3-fold to [Asn^3^, Lys^6^, Phe^13^]3–14-bombesin. Since the seven amino acids at the C-terminus of bombesin and its homologs are considered the biologically active site (Lin et al., 1995; König et al., 2015; Kakali et al., 2019) and these two BLPs share a highly similar structure at this region, it is suggested that both might cause myotropic contraction by binding to the bombesin receptors, while both BLPs only contain one amino acid substitution from Val^8^ to Thr^8^. Therefore, it is very likely that the enhanced myotropic potency of [Asn^3^, Lys^6^, Thr^10^, Phe^13^]3–14-bombesin resulted from this substitution. Litorin, a family of bombesins, with nine amino acids in length, was initially discovered from the skin of the Australian hylid frog, *Litoria aurea* (Zhou et al., 2017). With a short structure, litorin was found to have a higher potency than bombesin especially on rat urinary bladder (Anastasi et al., 1975). [Asn^3^, Lys^6^, Phe^13^]3–14-bombesin has an identical sequence (from Gln^5^ to Met^12^) to the C-terminal octapeptide of litorin. A previous study reported that the single substitution (Thr^5^ for Val^5^) in litorin could cause an increased contractile potency on the rat urinary bladder (Anastasi et al., 1975). Similarly, in comparison to [Asn^3^, Lys^6^, Phe^13^]3–14-bombesin, a higher potency of [Asn^3^, Lys^6^, Thr^10^, Phe^13^]3–14-bombesin on either the rat urinary bladder or uterus was observed in this study. It was speculated that the greater potency of [Asn^3^, Lys^6^, Thr^10^, Phe^13^]3–14-bombesin toward the urinary bladder and uterus may be attributed to the substitution from valine to threonine. Additionally, compared to litorin, ranatensin has two more amino acids at the N-terminus and normally shows lower contractile potency on the rat uterus (Anastasi et al., 1975). These two BLPs had an extended region at the N-terminus; therefore, it was assumed that both the length and the composition of amino acids of the N-terminus play determinant roles in the potency of BLPs.

To investigate the receptor selectivity, the rat urinary bladder and uterus contractile properties modulated by [Asn^3^, Lys^6^, Thr^10^, Phe^13^]3–14-bombesin and [Asn^3^, Lys^6^, Phe^13^]3–14-bombesin in the presence of BIM 23042, a well-established bombesin BB1 antagonist and BW2258U89, a selective bombesin BB2 antagonist, were examined. Of interest is that significant attenuations (70 %–80 %) were observed in either BB1 receptor antagonist or BB2 receptor antagonist treatment condition on the rat bladder, and a combined pre-treatment of BIM 23042 and BW2258U89 showed no significant difference from a single application of either BIM 23042 or BW2258U89. It indicated that [Asn^3^, Lys^6^, Thr^10^, Phe^13^]3–14-bombesin and [Asn^3^, Lys^6^, Phe^13^]3–14-bombesin had an affinity to both BB1 and BB2 receptors, and they exerted their contractile functions by binding to both BB1 and BB2 receptors on the rat bladder. However, BIM 23042 surprisingly did not considerable attenuate contractile effects on the rat uterus, while only BW2258U89 significantly reduced the effect. It implied that the contractile function of both BLPs on rat uterus smooth muscle preparations is mainly modulated by the BB2 receptor. This is probably due to the fact that the BB1 receptor is rarely expressed in rat uterus or BB2 receptor-mediated signaling is mainly responsible for bombesin-evoked contraction in rat uterus (Xiao and Reitman, 2016).

Additionally, both BLPs at a concentration of 1 μM maintained the contractile activity partially on the rat bladder in a combined pre-treatment of the BB1 antagonist and BB2 antagonist, which implied that some other types of receptors were likely involved in the contractile response to them. Previous studies reported that the orphan bombesin receptor, like the BB3 receptor with a high degree of homology to the BB1 and BB2 receptors, exists in mammals, which is responsible for regulating insulin release, food intake, blood pressure, heart rate, body temperature, and energy balance (Li et al., 2019). Therefore, the orphan bombesin receptor is also likely involved in mediating the contractile response of the rat urinary bladder to these two novel BLPs that may have different binding affinities/motions toward the orphan bombesin receptor. This finding is significant as no high-affinity native agonist to the BB3 receptor has been found until now. This may contribute to designing new potent and selective ligands for the orphan bombesin receptor. However, further studies are needed to prove if these two bombesins also serve as ligands for the orphan receptor BB3.

Research indicates that an underactive bladder condition in elderly patients with chronic medical or neurological diseases has been implicated in elevated post-void residual urine volumes and symptoms of frequency, nocturia, and urinary tract infections (Miyazato et al., 2013; Jiang and Kuo, 2017). Clinically, bethanechol (a muscarinic receptor agonist) and distigmine (an acetylcholinesterase inhibitor) are commonly used in the treatment of underactive detrusor and voiding dysfunction in the urinary tract. However, the severe side effects of these therapies, associated with the use of parasympathomimetics, have largely limited their efficacies and tolerability, and therefore, there is a need to find new treatments that can contract the bladder with minimal side effects (Jiang and Kuo, 2017). In such circumstances, bombesin and its relatives directly acting on bladder smooth muscle contractility are promising and may be beneficial for complete bladder emptying. In addition, research in the past 30 years revealed that overexpressed bombesin receptors (e.g., NMB and GRP receptors) in human tumors can be targeted successfully (Morgat et al., 2017; Reubi et al., 2002). A study conducted by Cescato et al. in 2008 has proven the ability of a bombesin analog, demobesin 1, in triggering the internalization of the BB1 receptor on human prostate cancer specimens (Cescato et al., 2008). Furthermore, Accardo et al. (2019) conducted a preliminary animal study in 2019 on prostate cancer xenograft mouse models and confirmed that bombesin-modified liposomal doxorubicin could effectively reduce tumor growth in comparison to plain liposomal doxorubicin. These studies suggest that bombesin and its analogs as targeting ligands may provide an effective and targeted drug delivery system for cancer therapeutics.

## 5 Conclusion

This study reported two native BLPs acting as an agonist for BB1 and BB2 receptors, isolated from the hybridogenic frog, *Pelophyla*x kl. *esculentus*. They have stimulant effects on smooth muscle contraction with high potencies. Preliminary investigation on horse erythrocytes and normal human cell line HMEC-1 indicated that these two BLPs did not demonstrate significant *in vitro* cellular toxicity. However, further toxicology investigations in living animals should be considered in the future study. These two novel BLPs provide a novel insight into the treatment of impaired bladder/uterus contractions such as voiding dysfunction and prolonged labor. Additionally, since the bombesin receptors are widely expressed in mammals, the therapeutic potential of BLPs to treat multiple diseases, for instance, cancer cell growth, blood pressure, gastrin release, and pancreatic secretion-associated disorders, are worthwhile to study in the future.

## Data Availability

The raw data supporting the conclusions of this article will be made available by the authors, without undue reservation.

## References

[B1] Accardo A. Mannucci S. Nicolato E. Vurro F. Diaferia C. Bontempi P. (2019). Easy formulation of liposomal doxorubicin modified with a bombesin peptide analogue for selective targeting of GRP receptors overexpressed by cancer cells. Drug Deliv. Transl. Res. 9, 215–226. 10.1007/s13346-018-00606-x 30569349

[B2] Anastasi A. Erspamer V. Endean R. (1975). Aminoacid composition and sequence of Litorin, a Bombesin-like nonapeptide from the skin of the Australian leptodactylid frog Litoria aurea. Experientia 31, 510–511. 10.1007/BF01932427 1140241

[B3] Bai B. Wang H. Xue Y. Wu Y. Zhou M. Wei M. (2012). The structures of four bombesins and their cloned precursor-encoding cDNAs from acid-solvated skin secretion of the European yellow-bellied toad, *Bombina variegata* . Peptides 36 (2), 221–229. 10.1016/j.peptides.2012.05.020 22687368

[B4] Baratto L. Duan H. Y. Macke H. Iagaru A. (2020). Imaging the distribution of gastrin-releasing peptide receptors in cancer. J. Nucl. Med. 61, 792–798. 10.2967/jnumed.119.234971 32060215

[B5] Bardin C. W. (1993). Recent progress in hormone research: Proceedings of the 1991 laurentian hormone conference, 48. Saint Louis: Elsevier Scienvier & Technology, 1–553.

[B6] Battey J. Benya R. V. Jensen R. T. Moody T. W. (2021). Bombesin receptors in GtoPdb v.2021.2. IUPHAR/BPS Guide Pharmacol. CITE 2021, 2. 10.2218/gtopdb/F9/2021.2

[B7] Bilusich D. Jackway R. J. Musgrave I. F. Tyler M. J. Bowie J. H. (2009). The host‐defence skin peptide profiles of Peron's Tree Frog Litoria peronii in winter and summer. Sequence determination by electrospray mass spectrometry and activities of the peptides. Rapid Commun. Mass Spectrom. 23, 2628–2636. 10.1002/rcm.4164 19642086

[B8] Blais K. SethiIustin J. Tabarean L. V. (2016). Gastrin-releasing peptide receptor mediates the excitation of preoptic GABAergic neurons by bombesin. Neurosci. Lett. 633, 262–267. 10.1016/j.neulet.2016.09.045 27693662 PMC5074900

[B9] Cescato R. Maina T. Nock B. Nikolopoulou A. Charalambidis D. Piccand V. (2008). Bombesin receptor antagonists may be preferable to agonists for tumor targeting. J. Nucl. Med. 49 (2), 318–326. 10.2967/jnumed.107.045054 18199616

[B10] Daum J. M. Davis L. R. Bigler L. Woodhams D. C. (2012). Hybrid advantage in skin peptide immune defenses of water frogs (Pelophylax esculentus) at risk from emerging pathogens. Infect. Genet. Evol. 12 (8), 1854–1864. 10.1016/j.meegid.2012.07.024 22940461

[B11] Davis T. P. Crowell S. Taylor J. Clark D. L. Coy D. Staley J. (1992). Metabolic stability and tumor inhibition of bombesin/GRP receptor antagonists. Peptides 13 (2), 401–407. 10.1016/0196-9781(92)90128-p 1329046

[B12] Fleischmann A. Waser B. Gebbers J. Reubi J. C. (2005). Gastrin-releasing peptide receptors in normal and neoplastic human uterus: Involvement of multiple tissue compartments. J. Clin. Endocrinol. Metab. 90 (8), 4722–4729. 10.1210/jc.2005-0964 15941862

[B13] Gonzalez N. Moody T. W. Igarashi H. Ito T. Jensen R. T. (2008). Bombesin-related peptides and their receptors: Recent advances in their role in physiology and disease states. Curr. Opin. Endocrinol. Diabetes Obes. 15, 58–64. 10.1097/MED.0b013e3282f3709b 18185064 PMC2631407

[B14] Hiroko O. (2000). H. Neuromedin B. Prog. Neurobiol. 62 (3), 297–312. 10.1016/S0301-0082(00)00004-6 10840151

[B15] Hirooka A. Hamada M. Fujiyama D. Takanami K. Kobayashi Y. Oti T. (2021). The gastrin-releasing peptide/bombesin system revisited by a reverse-evolutionary study considering Xenopus. Sci. Rep. 11, 13315. 10.1038/s41598-021-92528-x 34172791 PMC8233351

[B16] Jensen R. Battey J. Spindel E. Benya R. (2008). International union of pharmacology. LXVIII. Mammalian bombesin receptors: Nomenclature, distribution, pharmacology, signaling, and functions in normal and disease states. Pharmacol. Rev. 60, 1–42. 10.1124/pr.107.07108 18055507 PMC2517428

[B17] Jiang Y. Kuo H. (2017). Video-urodynamic characteristics of non-neurogenic, idiopathic underactive bladder in men – a comparison of men with normal tracing and bladder outlet obstruction. PLoS ONE 12 (4), e0174593. 10.1371/journal.pone.0174593 28376105 PMC5380335

[B18] Kakali D. Dibyanti M. Samarendu S. Shantanu G. (2019). HYNIC and DOMA conjugated radiolabeled bombesin analogs as receptor-targeted probes for scintigraphic detection of breast tumor. EJNMMI Res. 9, 25. 10.1186/s13550-019-0493-x 30887136 PMC6423188

[B19] Kilgore W. Mantyh P. Mantyh C. McVey D. Vigna S. (1993). Bombesin/grp-preferring and neuromedin b-preferring receptors in the rat urogenital system. Neuropeptides 24, 43–52. 10.1016/0143-4179(93)90039-d 8381528

[B20] König E. Bininda-Emonds O. R. Shaw C. (2015). The diversity and evolution of anuran skin peptides. Peptides 63, 96–117. 10.1016/j.peptides.2014.11.003 25464160

[B21] Ladenheim E. E. (2013). “Chapter 142 - bombesin,” in Handbook of biologically active peptides. Editor Kastin A. J.. Second Edition (Boston: Academic Press), 1064–1070.

[B22] Li M. Liang P. Liu D. Yuan F. Chen G. Zhang L. (2019). Bombesin receptor subtype-3 in human diseases. Arch. Med. Res. 50 (7), 463–467. 10.1016/j.arcmed.2019.11.004 31911345

[B23] Lin J. T. Coy D. H. Mantey S. A. Jensen R. T. (1995). Comparison of the peptide structural requirements for high affinity interaction with bombesin receptors. Eur. J. Pharmacol. 294 (1), 55–69. 10.1016/0014-2999(95)00510-2 8788416

[B24] Marenah L. Flatt P. R. Orr D. F. McClean S. Shaw C. Abdel-Wahab Y. H. (2004). Skin secretion of the toad *Bombina variegata* contains multiple insulin-releasing peptides including bombesin and entirely novel insulinotropic structures. Biol. Chem. 385 (3-4), 315–321. 10.1515/BC.2004.027 15134346

[B25] Marquez C. Treston A. Moody E. Jakowlew S. Moody T. (2000). The metabolism of BW2258U89, a GRP receptor antagonist. Neuropeptides 34 (2), 108–115. 10.1054/npep.2000.0798 10985927

[B26] Miyazato M. Yoshimura N. Chancellor M. (2013). The other bladder syndrome: Underactive bladder. Rev. Urol. 15 (1), 11–22. 23671401 PMC3651538

[B27] Morgat C. MacGrogan G. Brouste V. Vélasco V. Sevenet N. Bonnefoi H. (2017). Expression of gastrin-releasing peptide receptor in breast cancer and its association with pathologic, biologic, and clinical parameters: A study of 1, 432 primary tumors. J. Nucl. Med. 58 (9), 1401–1407. 10.2967/jnumed.116.188011 28280221

[B28] Ohki-Hamazaki H. (2016). “Subchapter 22B - neuromedin B,” in Handbook of hormones. Editors Takei Y. Ando H. Tsutsui K. (San Diego: Academic Press), 193–e122B.

[B29] Pukala T. L. Bertozzi T. Donnellan S. C. Bowie J. H. Surinya‐Johnson K. H. Liu Y. (2006). Host‐defence peptide profiles of the skin secretions of interspecific hybrid tree frogs and their parents, female Litoria splendida and male Litoria caerulea. FEBS J. 273 (15), 3511–3519. 10.1111/j.1742-4658.2006.05358.x 16824041

[B30] Pukala T. L. Bowie J. H. Maselli V. M. Musgrave I. F. Tyler M. J. (2006). Host-defence peptides from the glandular secretions of amphibians: Structure and activity. Nat. Prod. Rep. 23, 368–393. 10.1039/b512118n 16741585

[B31] Ramos-Alvarez I. Moreno P. Mantey S. A. Nakamura T. Nuche-Berenguer B. Moody T. W. (2015). Insights into bombesin receptors and ligands: Highlighting recent advances. Peptides 72, 128–144. 10.1016/j.peptides.2015.04.026 25976083 PMC4641779

[B32] Reubi J. Wenger S. Schmuckli-Maurer J. Schaer J. Gugger M. (2002). Bombesin receptor subtypes in human cancers: Detection with the universal radioligand 125I-[D-TYR6, β-ALA11, PHE13, NLE14] bombesin (6–14). Clin. Cancer Res. 8 (4), 1139–1146. 11948125

[B33] Salahudeen M. Nishtala P. (2017). An overview of pharmacodynamic modelling, ligand-binding approach and its application in clinical practice. Saudi Pharm. J. 25 (2), 165–175. 10.1016/j.jsps.2016.07.002 28344466 PMC5355565

[B34] Spindel E. (2013). Handbook of biologically active peptides. Second Edition. (Cambridge, MA: Academic Press), 46, 326–330.

[B35] Spindel E. R. (2006). “Amphibian bombesin-like peptides,” in Handbook of biologically active peptides. Editor Kastin A. J. (Amsterdam: Elsevier), 277–281.

[B36] Spindel E. R. Giladi E. Segerson T. P. Nagalla S. (1993). Bombesin-like peptides: Of ligands and receptors. Recent Prog. Horm. Res. 48 (1), 365–391. 10.1016/b978-0-12-571148-7.50017-8 8382830

[B37] Williams J. A. (2015). Bombesin. Pancreapedia: Exocrine Pancreas Knowledge Base, 1–6. 10.3998/panc.2013.5

[B38] Xiao C. Reitman M. L. (2016). Bombesin-like receptor 3: Physiology of a functional orphan. Trends Endocrinol. Metab. 27 (9), 603–605. 10.1016/j.tem.2016.03.003 27055378 PMC4992652

[B39] Zhou X. W. Ma C. B. Zhou M. Zhang Y. N. Xi X. P. Zhong R. M. (2017). Pharmacological effects of two novel bombesin-like peptides from the skin secretions of Chinese piebald odorous frog (Odorrana schmackeri) and European edible frog (Pelophylax kl. esculentus) on smooth muscle. Molecules 22 (10), 1798. 10.3390/molecules22101798 PMC6151381 29065544

