# Supplementary material for: Two novel bombesin-like neuropeptides from the skin secretion of Pelophylax kl. esculentus: Ex vivo pharmacological characterization on rat smooth muscle types
Source: Front Mol Biosci. 2022 Sep 29;9:953974. doi: 10.3389/fmolb.2022.953974 (PMC9560764; doi:10.3389/fmolb.2022.953974)
Supplement: Supplementary file 1 [file DataSheet1.docx]

**Supplementary Figure 1**. RP-HPLC chromatogram monitored at 214 nm (a) and full scan mass spectrum (b) of purified peptide [Asn^3^, Lys^6^, Thr^10^, Phe^13^]3-14-bombesin. The acetonitrile gradient is indicated by solid blue line. Mass peaks of singly- and doubly- charged ions in the mass spectrum are indicated by arrows.

**Supplementary Figure 2**. RP-HPLC chromatogram monitored at 214 nm (a) and full scan mass spectrum (b) of purified peptide [Asn^3^, Lys^6^, Phe^13^]3-14-bombesin. The acetonitrile gradient is indicated by solid blue line. Mass peaks of singly-, doubly- and triply- charged ions in the mass spectrum are indicated by arrows.

**Supplementary Figure 3**. NMR proton spectra recorded at 400 MHz and 25 ˚C for purified peptide (A) [Asn^3^, Lys^6^, Phe^13^]3-14-bombesin and (B) [Asn^3^, Lys^6^, Thr^10^, Phe^13^]3-14-bombesin in deuterium oxide (D_2_O).

**Supplementary Figure 4.** Nucleotide and translated open-reading frame amino acid sequences of cloned cDNA encoding the biosynthetic precursors of **(a)** [Asn^3^, Lys^6^, Thr^10^, Phe^13^]3-14-bombesin and **(b)** [Asn^3^, Lys^6^, Phe^13^]3-14-bombesin. The coding sequence regions corresponding to putative signal peptides and mature peptides are double-underlined and single-underlined, respectively. Asterisks indicate stop codon.

**Supplementary Figure 5. (a)** Alignment of open reading frame of [Asn^3^, Lys^6^, Thr^10^, Phe^13^]3-14-bombesin and [Asn^3^, Lys^6^, Phe^13^]3-14-bombesin; **(b)** Comparison of amino acid sequences of prepropeptides from the skin of *Pelophylax* kl. *esculentus*. The amino acid sequence of the mature peptides is underlined. Conserved sequences are highlighted in yellow. The amino acid substitution in mature domain is highlighted in green. Note that nucleotide substitution sites occur throughout the sequences.

**Supplementary Figure 6.** LCQ MS/MS sequencing results of **(a)** [Asn^3^, Lys^6^, Thr^10^, Phe^13^]3-14-bombesin and **(b)** [Asn^3^, Lys^6^, Phe^13^]3-14-bombesin from HPLC fractions indicated in Figure 1.
